# Environmental education positively impacts the perceptions of learners towards bats in schools in a low socio-economic area in South Africa

**DOI:** 10.1371/journal.pone.0335652

**Published:** 2025-12-19

**Authors:** Veli Monday Mdluli, Alexandra Howard, Emile Bredenhand, Ara Monadjem, Bekithemba Dube, Adrinajoro Rakotoarivelo, Peter John Taylor

**Affiliations:** 1 Afromontane Research Unit, Department of Zoology & Entomology, University of the Free State, Phuthaditjhaba, South Africa; 2 Department of Education, University of the Free State, Phuthaditjhaba, South Africa; 3 Department of Biological Sciences, University of Eswatini, Kwaluseni, Eswatini; 4 Mammal Research Institute, Department of Zoology and Entomology, University of Pretoria, Hatfield, Pretoria, South Africa; Saint Mary's University, CANADA

## Abstract

Bats are amongst the most important ecosystem service providers in the world. This is in part due to their diversity as they are the second largest group of mammals with a widespread global distribution. Despite this, most cultures around the world have negative stereotypes towards bats, largely influenced by myths and beliefs stemming from misconceptions about them. In this study, we aimed to assess the impact of environmental education on enhancing awareness and positive perceptions towards bats and their natural ecosystem services by school learners of ages 11–18 years old in QwaQwa, in the eastern Free State Province of South Africa. Our study addressed negative stereotypes about bats through informed and interactive engagement of scientific and public communication in schools with the use of bat awareness classes, posters, a children’s book on bats, and engagement in bat research revolving around bat boxes installed at each school. We used a standardized questionnaire to assess individual attitudes towards bats pre- and post-intervention, targeting for each survey approximately 200 learners from two primary and one secondary schools in QwaQwa. The perceptions of students were divided into six categories namely scientistic, positivistic, behavioral negativistic, emotional negativistic, cognitive negativistic and myth categories. The intervention resulted in a significant change in negative perceptions of students towards bats for all categories, becoming more positive. We also found gender and age to be important influencers of perceptions for some of the categories. Considering the findings, we argued that intervention through environmental education can be a useful tool for changing negative perceptions of learners towards bats in local South African communities.

## Introduction

Human-wildlife conflict results in the persecution of wild animals across the globe. Such conflict frequently stems from threats that wild animals pose to human safety and wellbeing such as food and property [1,2]. This phenomenon usually results in aggressive and retaliative behaviour from humans towards wildlife which is often fatal to the species of concern [3]. This, in turn, has negative implications on the conservation of these species [4]. The main driver behind human-wildlife conflict is the human perception of wildlife risk, which is often exaggerated compared to the actual risk [5,6]. Perceptions constitute subjective beliefs and emotions influenced by cultural, social, religious and individual factors, including personal experiences, media influence, and cultural norms [6–8], meaning that the perceptions that people have towards wildlife can determine the success or failure of species conservation initiatives. Since negative perceptions towards wildlife may lead to harming or killing of animals and destruction of their habitat [3,4], it is important to cultivate positive perceptions towards wildlife. This is especially important for children whose perceptions of wildlife today may influence how they interact with biodiversity in the future. Hence, the focus should not only be on shifting perceptions of adults but should also be inclusive of children.

One of the groups of animals which humans often have a negative perception towards, is that of bats. Bats are small mammals in the order Chiroptera comprising 1,500 extant species and forming the second largest mammalian order [9,10], thus accounting for 20% of all mammalian species in the world [11]. Not only are they important due to their widespread distribution, occupying every continent except for Antarctica, but bats have also been shown to have great ecological and economic roles [12–16]. Ecosystem services provided by bats include pollination, seed dispersal and pest insect suppression [17]. Pest insect suppression has been estimated for only a small number of crops, and only in a few regions of the world, but even so, the economic savings are enormous [12,15,16,18–20]. Despite this, bats continue to suffer from a bad image [21], perhaps linked to the often overexaggerated belief that they are instrumental in the spread of zoonotic diseases [22–26]. Despite their significance, many bat populations are globally threatened [9,17], with human activities such as land-use changes resulting from agriculture, logging, livestock ranching, urbanization, hunting and persecution being the main culprits [9,27–29].

Bats have been shown to be one of the animal groups that humans tend to have phobias of, together with snakes, mice and spiders [30,31]. The long history of negative stereotypes towards bats can be accredited to misconceptions which have been perpetuated through myths. In some cultures, bats are believed to be associated with witchcraft due to their nocturnal nature [32]. They are also often depicted as vampires in art and literature, despite the fact that there are only three species of blood-feeding bats, which rarely consume human blood and do not occur in Africa [33]. They are also disdained for occupying the roofs of houses, causing annoyance to residents through chattering sounds at night and through accumulation of urine and faeces [34]. Some farmers also view bats as pests due to foraging on fruit tree farms, hence lowering potential yield from those plantations [9,35]. Bats have also been implicated in transmission of zoonotic diseases such as coronaviruses, lyssaviruses, and filoviruses to humans [36]. These negative stereotypes have further escalated due to the belief that bats were responsible for the COVID-19 pandemic [37–39]. In fearing and hating bats for potential disease transmission, people overlook that the transfer of zoonotic diseases from animals to people mostly occurs when people handle animals, for example to kill them. Since the outbreak of the COVID pandemic, bats have been subject to stigmatizations in places such as in Indonesia where hundreds of bats have been killed due to the fear of the transmission of the COVID-19 virus [40]. However, people who were aware of the importance of bats in ecosystem functioning were less likely to have negative perceptions towards bats, even during the pandemic [40]. Not all cultures view bats negatively. A study showed that some pastoralists in Namibia believed that bats were a sign of good luck and good rains [41]. Some cultures in China also believe that bats are a sign of good luck because the word “bat” is pronounced the same as that of “fortune” in Mandarin Chinese [42].

Hence, environmental education may be key to changing perceptions. It has already been successfully used to change attitudes towards wildlife species previously considered harmful to humans [43]. For example, environmental education has proved to be effective in reducing negative attitudes toward owls in South Africa [44], and in mediating conflict with elephants in Zimbabwe [45]. Educational awareness classes come in different forms, including the use of presentations, workshops and citizen science projects. The effectiveness of the intervention lies in its ability to equip people with knowledge, skills, and values needed for informed environmental decision-making. Environmental education, however, must be informative and relevant to its target audience. It is therefore crucial that before the implementation of such a tool, gaps in knowledge have already been addressed. Although environmental education has become a popular tool to spread awareness about the importance of wildlife, the attitudes and perceptions of the people who have received the education are rarely assessed. There is also a dearth of studies that quantify the degree to which environmental education can influence the perception of the people in question [46], particularly in Africa.

Therefore, we aimed to ascertain the perception of sixth- and eighth grade learners towards bats and determine whether environmental education could be used as an effective tool to change negative perceptions that school learners might have about bats. Studies focusing on perceptions of people towards bats in South Africa are lacking despite the common interaction of bats and people in this region. We focused on schools located in QwaQwa, a low-income area [47] where most residents are likely to be encountering bats as most lack the resources to ensure that their housing infrastructure is not accessible to bats. QwaQwa is one of the areas located in the Maloti-Drakensberg region where endemic bats have been recorded, making it critical that the people in the area know the value of bats. Based on a similar study by Williams et al. (2021) focusing on owls, we hypothesized that educational intervention would result in reduced negative perceptions towards bats.

## Methods

### Study site

The study was conducted in Phuthaditjhaba, a town in QwaQwa. This area is located in the eastern part of the Free State Province in South Africa, near the Kingdom of Lesotho. This region is under the Maloti-A-Phofung Local Municipality and the Thabo Mofutsanyane District and is situated on the foothills of the Maloti-Drakensberg mountains [48]. The estimated population in Maloti-A-Phofung is 335,784 people [49]. This region was established as a homeland for the Basotho people in the 1970s and was reincorporated into the Free State Province in 1994, following the end of apartheid [50].

Despite QwaQwa’s beautiful scenery, which includes the Golden Gate Highlands National Park, and several educational institutions such as the QwaQwa campus of the University of the Free State, the region faces several challenges, including high levels of poverty, unemployment, and crime [51], all of these having been worsened by the recent COVID-19 pandemic. Additionally, access to basic services such as clean water and electricity is limited in this region.

### Sampling participants

**Fig 1:**
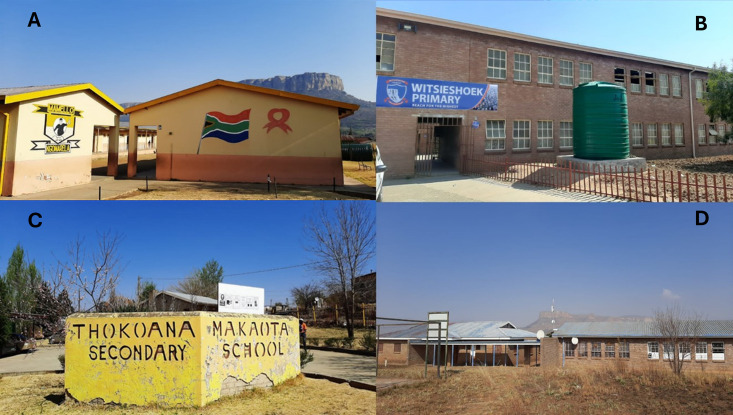
The four schools where the survey was conducted: A) Mamello Primary; B) Witsieshoek Primary; C) Thokoana Makaota Secondary; D) Bluegumbosch Secondary, all located in QwaQwa, Free State Province of South Africa.

**Fig 2:**
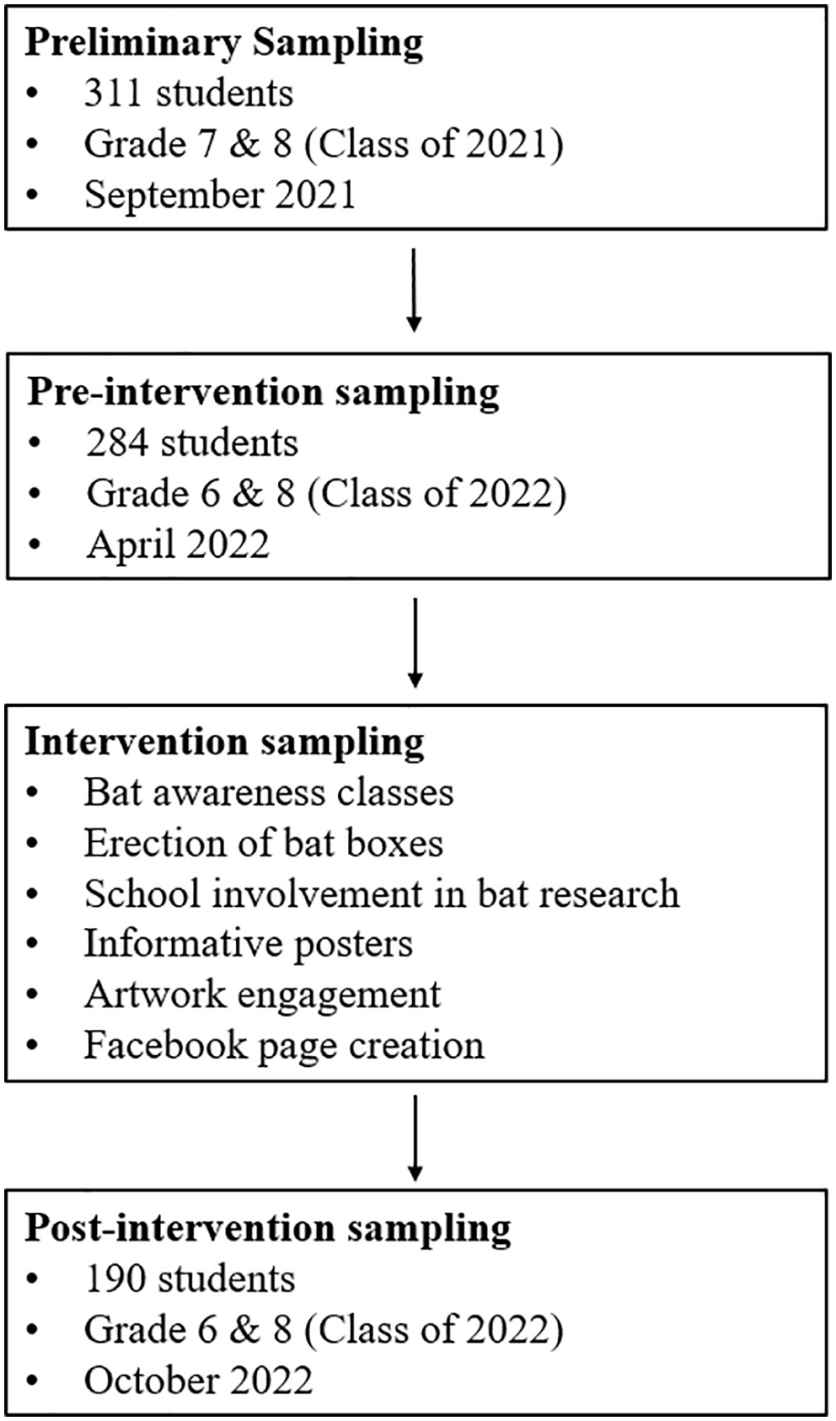
A schematic outlining the sampling process for the study.

The study was conducted from the 20^th^ of September 2021 and ended on the 14^th^ of October 2022. Two high schools (Thokoana Makaota Secondary and Bluegumbosch Secondary School) and two primary schools (Mamello Primary and Witsieshoek Primary School) (Fig 1) were initially selected in QwaQwa for data collection. These schools were randomly selected, although this selection favored schools that were not too far from the University of the Free State, QwaQwa campus as per logistic requirements. During the first stage of the study (September 2021), a preliminary study was conducted in four schools, focusing on seventh grade learners in the primary schools and eighth grade learners in the high schools (with ages ranging from 11 to 13 years and 13–18 years of age respectively). This was done to test the suitability of our methodology before the actual study began. The actual study commenced in 2022 with both pre-intervention and post-intervention sessions taking place the same year, as shown in Fig 2. For this survey, we focused on sixth and eighth grade learners, because they were at a stage where they understood the concept of answering questionnaires while being young enough to still be considered children. Pre-intervention survey was defined as the handing out of questionnaires to learners before learners were exposed to any bat awareness environmental education (Fig 2). Post-intervention was defined as surveying learners through questionnaires after a bat awareness educational session, about six months after the pre-intervention. Complete data collection could only be done in three schools (unfortunately, the post-intervention exercise at Bluegumbosch Secondary School had to be abandoned due to unforeseen circumstances).

### Questionnaire structure

We utilized a standardized questionnaire designed to assess people’s attitudes towards bats by Perez et al. (2021). The questionnaire comprised of 35 statements (which students could agree or disagree with) which were divided into six categories namely scientistic, positivistic, emotional negativistic, behavioral negativistic, cognitive negativistic and myths (Table 1). These categories were formulated by Perez et el. (2021), but we did make statements relatable to the learners. The scientistic section comprised statements which assessed whether the learners were willing to learn about bats and if they were willing to share their knowledge with others. The statements of the positivistic section assessed whether learners knew about the ecological and economic importance of bats. The emotional negativistic section comprised statements assessing the learners’ emotions towards bats, such as whether they feared them. The behavioral negativistic assessed whether they felt bats should be removed from the environment, while the cognitive negativistic assessed whether learners perceived bats as being harmful to humans. The myth section assessed whether learners believed in myths. Since the perception categories used in the study were developed based on a framework adapted from existing literature outside our study area, we refined the categories through feedback from local students in focus groups, ensuring they were both relevant and comprehensive for the target population. The questionnaire statements were originally developed in English and subsequently translated into seSotho, the local language, to mitigate potential limitations associated with varying levels of English proficiency among learners. While learners in the selected grades are generally expected to have competency in English, by translating the statements into seSotho, we ensured clarity, inclusivity, and accurate comprehension of the questionnaire and intervention. Due to the study being conducted after the COVID-19 pandemic, a statement on whether learners believed COVID came from bats was also added to the statements formulated by Perez et al. (2021). Each of the six categories comprised a varying number of statements (S1 Appendix). The questionnaire was initially in a Likert-scale format with each of the statements having a set of choices rating their response from 1 (Strongly disagree) to 5 (Strongly agree), with a score of 3 representing a neutral response (S2 Appendix).

**Table 1:** Categories of learners’ perceptions towards bats and the attitudes assessed before and after the educational intervention.

Category	Perception/attitude assessed
Scientistic	The learner’s interest, curiosity, and engagement in scientific activities related to bats
Positivistic	The learner’s belief in the positive contributions of bats to the ecosystem, agriculture, and human well-being, as well as the importance of protecting and conserving bats
Emotional negativistic	The learner’s negative emotional responses and perceptions towards bats, including fear, aversion, and negative aesthetic judgments
Behavioural negativistic	The learner’s advocacy for or endorsement of actions that involve harmful or aggressive behaviour towards bats
Cognitive negativistic	The learner’s beliefs or perceptions that attribute negative consequences to bats, particularly in relation to agricultural activities, environmental contamination, and potential dangers to humans and animals
Myths	The learner’s beliefs or narratives about bats that are not based on scientific evidence or factual knowledge

### Sampling design

In September of 2021, learners’ perceptions were assessed using the standardized questionnaire in the four schools as part of a preliminary survey. Upon completion of the questionnaire, learners were then given a 40-minute bat awareness presentation, using slides presentation to convey scientific knowledge that sought to inform learners about the nature and behavior of bats, the critical roles that bats play in the ecosystem, and to challenge common misconceptions about bats. The importance of bat conservation, especially with regards to the two bat species endemic to the Maloti-Drakensberg mountains, namely *Cistugo lesueuri* and *Laephotis* cf*. wintoni* [52] was also a major aspect of the presentation. Learners were allowed to ask questions at any point of the presentation. The content and format of the presentation was deemed suitable for the learners by the Faculty of Education at the University of the Free State. In addition, we obtained a Human Research ethics permit (number: UFS-HSD2021/0914/21). Consent for student participation in this study was obtained from both the students and their parents or guardians. Each student and at least one parent or guardian were required to sign separate consent forms to confirm their approval. We also obtained an animal ethics permit (number: UFS-AED2021/0029/21) from the Animal Research Ethics Committee from the University of the Free State. Once the class was finished, two posters containing the taught information were also donated to each school to allow learners to refer to them whenever they needed to. The educational materials, including the slide presentation and the information provided on posters, were specifically tailored to the cultural context of the QwaQwa community. The myths, such as the local belief that bats eat human hair, were addressed directly in the materials, presenting scientific evidence to counter these misconceptions. Additionally, the educational content incorporated local cultural references and examples, such as the role of bats in agriculture and pest control, making the information more accessible and engaging. The materials were even translated from English into seSotho, the common local language, ensuring that the learners could fully understand and relate to the content. A Facebook page entitled QQ Bat Education Project on the link (https://www.facebook.com/profile.php?id=100076140791500) was set up to allow students to engage with conductors of the study and to ask questions. This page was accessible to all the students in this study, as well as to all members of the public.

**Fig 3:**
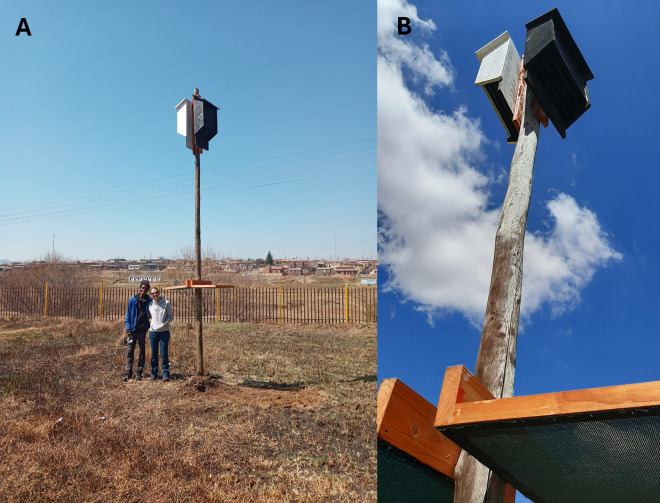
Examples of pairs of black and white bat boxes erected at the schools as part of the school and student engagement. **(A)** Bat boxes at Bluegumbosch Secondary School; **(B)** Close-up of bat boxes at Mamello Primary School.

Less than a week after presentations were made, four bat boxes were erected in each of the two high schools, and two bat boxes were erected in each of the two primary schools (Fig 3). These bat boxes were used as a citizen science tool to engage and involve learners. Although the bat boxes were part of another study assessing the diet of bats, their installation and monitoring allowed the students to engage with the researchers and observe the bats roosting on the premises of each school. Part of the intervention also included an artwork competition for students, where they could draw anything related to bats. The winning artwork was featured in a children’s fictional book by the first author, set in QwaQwa, which includes educational facts promoting bat conservation (see S1 File). The book itself was an intervention strategy although it had not been published by the time of the post-intervention survey. The pre-intervention survey was conducted in April of 2022 in the four schools with a new group of learners (Fig 2). This survey focused on sixth and eighth grade learners. The post-intervention survey was conducted six months later on the same learners who had been part of the study that year. For the pre-intervention survey, which involved learners completing a questionnaire before any educational activities, responses from 284 learners were used for analysis. In contrast, the post-intervention survey analyzed responses from 192 learners. The 32.4% reduction in responses for the post-intervention survey was due to some students being absent on the day the survey was conducted.

### Data analysis

Data from the three schools (Mamello Primary, Witsieshoek Primary and Thokoana Makaota Secondary) was used to test whether the educational intervention had changed the perceptions of learners with regards to the six categories defined above. For testing, we combined the “strongly agree” and “agree” responses into an “agree” category. The same was done for the “strongly disagree” and “disagree” responses. This left us with three responses: “agree”, “neutral” and “disagree”. We then used the clmm (Cumulative Link Mixed Model) function from the “ordinal” package [53] in R studio version 4.3.0 [54] to run mixed ordinal regression analysis assessing the influence of predictor variables on the ordinal response variable consisting of the three categories namely, “agree,” “disagree,” and “neutral”. This method was chosen due to the ordinal nature of the response variable. The mixed ordinal regression models comprised of (school) and (grade) as random variables while intervention (before and after), gender (male and female) and (age) were the predictor variables. To assess the significance of predictors, p-values were calculated, and effect sizes were evaluated using odds ratios derived from the model coefficients. The Akaike’s Information Criterion (AIC) was used to compare models from each of the categories. The model with the lowest AIC value was selected as the best model, while those with ΔAIC less than 2 were deemed to have equal support [55,56]. Plots were created using ggplot2 [57] also in R studio version 4.3.0 [54].

## Results

**Fig 4:**
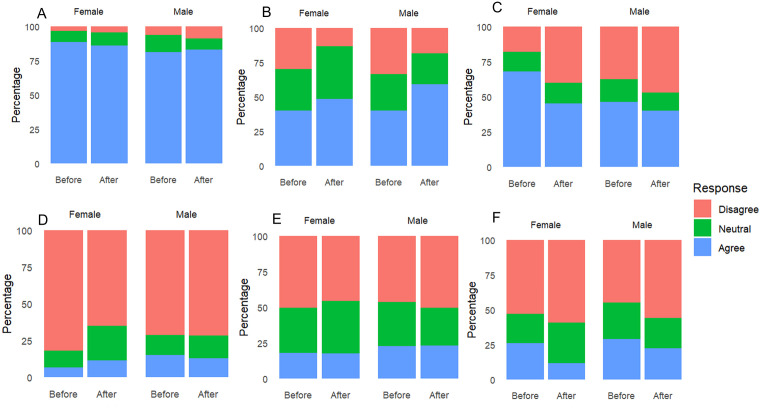
Percentage of responses for male and female learners by (A) scientistic statements, (B) positivistic statements, (C) emotional negativistic statements, (D) behavioural negativistic statements, (E) cognitive negativistic statements, (F) myth statements at the three schools before and after the educational intervention. The blue, green and red colours represent the proportions of learners who agree, remained neutral and disagreed respectively. For the scientistic (A) and positivistic categories **(B)**, agree responses (blue) are pro-bats whilst disagree responses (red) are anti-bat. For all the remaining categories **(C-F)**, the agree responses (blue) are anti-bat whilst the disagree responses (red) are pro-bat.

In this study, after excluding respondents who did not report their gender, a total of 284 students participated in the survey prior to the intervention, comprising 149 females (52.5%) and 135 males (47.5%) (S3 Appendix). Following the intervention, 190 students completed the survey, including 109 females (57.4%) and 81 males (42.6%) (S3 Appendix). The best performing models for the six perception categories identified “intervention” as a significant factor influencing changes in student perceptions of bats (Table 2). This suggests that our educational intervention positively impacted the learners’ attitudes toward bats. Fig 4 depicts the percentage of responses (agree, disagree or neutral) for male and female learners by scientistic (A), positivistic (B), emotional negativistic (C), behavioural negativistic (D), cognitive negativistic (E) and myth (F) categories. With regards to the different perception categories, responses to scientistic statements were mostly positive before our educational intervention. Despite this, our intervention still resulted in even higher positive perceptions amongst students for this category (Fig 4a). Age was also a significant factor in perceptions of students with increase in age showing more positive responses for the scientistic category (Table 2a). For the positivistic category, fewer students initially agreed that bats were beneficial for ecosystem functioning and that bat conservation is important compared to after the intervention (Fig 4b). A significant influence on perceptions was found in males for the positivistic category, where male learners showed a greater increase in agreement with statements about the ecological and economic importance of bats following the intervention compared to females (Table 2b).

**Table 2:** Results of mixed ordinal regression analysis assessing the influence of predictor variables on the ordinal response variable for the six perception categories with the estimates, standard errors, z-values, and p-values provided for each predictor variable. The predictor variables were intervention (before and after), gender (male and female) and age (younger and older). Statistically significant results are indicated by asterisks (*).

Category	Predictor	Estimate	Std. Error	z value	Pr (>|z|)	Significance
(A) Scientistic	InterventionAfter	0.364	0.123	2.960	0.00308	**
	GenderMale	0.4475	0.1021	4.384	1.16e-05	***
	AgeYounger	−0.3312	0.164	−2.019	0.04344	*
(B) Positivistic	InterventionAfter	−0.21247	0.07796	−2.725	0.00642	**
	GenderMale	−0.27673	0.05872	−4.713	2.45e-06	***
(C) Emotional negativistic	InterventionAfter	0.7527	0.1448	5.198	2.01e-07	***
	GenderMale	0.543	0.1059	5.125	2.98e-07	***
(D) Behavioral	InterventionAfter	0.5237	0.1401	3.739	0.000185	***
	GenderMale	−0.3055	0.1069	−2.857	0.00427	**
(E) Cognitive negativistic	InterventionAfter	0.37764	0.09009	4.192	2.77e-05	***
	GenderMale	−0.19809	0.0677	−2.926	0.00343	**
	AgeYounger	0.22764	0.10859	2.096	0.03605	*
(F) Myths	InterventionAfter	0.54742	0.10415	5.256	1.47e-07	***
	GenderMale	−0.14986	0.08067	−1.858	0.0632	
	AgeYounger	0.20018	0.13102	1.528	0.1265	

Significance codes: *** p < 0.001 ** p < 0.01 * p < 0.05.

For emotional negativistic views, both intervention and gender were significant predictors of learners’ perceptions (Table 2c). The intervention resulted in reduced negative emotional responses (e.g., fear of bats), with males showing less negative emotional response to bats compared to females (Fig 4c). For behavioural negativistic statements, intervention and gender also significantly influenced learners’ perceptions (Table 2d). The intervention reduced agreement with statements advocating for the removal of bats from the environment. More males advocated for removal of bats in the environment compared to females (Fig 4d).

For cognitive negativistic views, intervention, gender, and age were significant factors influencing learners’ perceptions of bats (Table 2e). Females showed a notable improvement in their perceptions following the intervention (Fig 4e), while older learners tended to have more negative responses, suggesting that age may influence pre-existing biases or beliefs about bats. Regarding myths, there was a noticeable reduction in the number of learners who believed in myths about bats in the post-intervention, indicating that the intervention effectively influenced their perceptions (Fig 4f). However, age and gender were not significant predictors for this category (Table 2).

## Discussion

The findings of this study revealed that learners initially had higher negative perceptions of bats before any intervention or environmental education (S3 Appendix). This negativity could be attributed to a lack of knowledge and prevalent misinformation about bats. Cohen (1973) found that high school learners with greater access to environmental information exhibited more positive attitudes compared to those with limited information, underscoring the critical role of knowledge in shaping perceptions and attitudes [58]. Similarly, other studies have highlighted the importance of environmental knowledge in influencing attitudes and behaviours [59,60]. Specifically, increased awareness and understanding of bats have been shown to correlate positively with improved attitudes toward them [32]. Hence, our study supports the findings of previous studies, demonstrating the effectiveness of this intervention tool in this region of southern Africa. The results further demonstrated that educational interventions could play a key role in reshaping young learners’ perceptions and attitudes toward bats. The study revealed that learners were eager to learn about bats and actively participate in classes focused on them. Notably, the negative beliefs they initially held about bats did not deter them from wanting to acquire knowledge or share what they learned with others, as reflected in their responses to the scientistic statements. This enthusiasm is particularly significant, as it provides learners with the opportunity to obtain accurate information, enabling them to make informed decisions about whether a species should be feared, or it is just misunderstood. As anticipated, the study also found that a low number of learners were aware of bats’ economic and ecological importance, as reflected in their responses to the positivistic statements. This lack of knowledge likely contributed to the overall negative perceptions of bats [21]. This suggests the need for the role of bats ecologically and economically to be made known to people and if possible, be demonstrated practically. A practical approach to demonstrating the significance of bats could enhance the impact of educational interventions. For example, taking students to fruit farms to witness firsthand the damage caused by pests and the potential financial losses could effectively highlight the ecological and economic benefits of bats in pest control. Additionally, excursions to local bat roosts, promoting the protection of cave roosts in QwaQwa, collaborating with rehabilitation centres, and organizing citizen science “bat walks” could all serve as engaging ways to foster interest in bats. Virtual tours and documentaries could also complement these efforts, offering an interactive and informative way to connect people with bat conservation. Interventions that give people a hands-on and personal experience, have been shown to change people’s perceptions about bats even more effectively [61–63]. The use of bat boxes as part of our intervention not only allowed participants to engage directly with a tangible conservation tool but also provided opportunities to observe bats in a safe and controlled manner. This practical involvement can foster a sense of stewardship, enhance understanding of bats’ ecological roles, and demystify common misconceptions by replacing abstract knowledge with real-world interaction. Behavioural responses showed minimal change. However, this was not a concern, as the initial responses were already highly positive, indicating that learners were not inclined to harm bats. This suggests that the intervention did not need to focus on discouraging harm, but rather on increasing awareness of the importance of bats. Regarding cognitive negativistic views, the lack of significant change could be attributed to the absence of a practical demonstration showing the importance of bats to the environment and their harmlessness to most human properties such as cars. The high number of neutral responses indicates a knowledge gap about bats. In contrast, emotional negativistic views showed a noticeable shift from negative to more positive perceptions. The lack of aggression towards bats, demonstrated by learners during the intervention, likely contributed to this change in emotional response.

Our study showed that educational intervention can be effective in changing perceptions of learners towards bats. The study showed that with proper education it is possible to undo negative stereotypes towards bats. The extent to how effective an intervention may be is also dependent on the type of intervention [44]. The success in changing learners’ perceptions towards bats may have been influenced by their observation of the authors conducting research on bats without encountering any of the negative myths, such as the belief that bats eat hair or bring ill omens—common local misconceptions. By witnessing the researchers engage with bats in a positive and safe manner, some learners likely reassessed their views, leading to a shift from agreeing with these myths to disagreeing with them. This aligns with findings from bat-watching tourism, where firsthand experience with bats has been shown to positively influence people’s attitudes [64,65]. These observations affirm that direct witnessing is a powerful tool in changing perceptions during educational interventions.

From this study, we gathered that approximately 20% of learners, both before and after the intervention, believed that bats caused the COVID 19 pandemic while approximately 50% did not. The lack of change in this perception following our intervention suggests that deeply held beliefs or misinformation about bats and disease transmission may be resistant to short-term educational efforts highlighting the need for more targeted, repeated, or context-specific interventions to effectively address misconceptions and promote accurate understanding of the role of bats in public health. The learners from the QwaQwa community also held a belief that bats eat human hair, which contributed significantly to their negative attitudes towards bats. This myth, previously unknown to the researchers, was not part of the questionnaire but was verbally confirmed by the learners during interactions. It was a belief passed down from adults, particularly parents, to encourage children to stay indoors after dark and to promote good behaviour. Even though no learner had ever witnessed a bat eating hair and there was no evidence to support the myth, it remained widespread across all the schools surveyed. This illustrates how deeply cultural and social beliefs can influence attitudes towards species, even without supporting evidence. It is worth noting that we focused on a localized sample in QwaQwa, and this provided valuable insights into the cultural and social factors influencing perceptions of bats in this community. However, the findings should be interpreted cautiously when extrapolated to other regions. Nevertheless, similar challenges—such as the prevalence of myths and lack of access to environmental education—are common in many rural and traditional societies globally, suggesting that the lessons learned here may have broader relevance with appropriate cultural adaptations. Furthermore, while promoting a positive image of bats is important for conservation, it is also crucial to acknowledge potential public health risks, as bats can carry diseases such as rabies. In our study, this concern was mitigated because the intervention explicitly addressed the dangers of handling bats irresponsibly and emphasized the importance of leaving bats undisturbed. Therefore, the positive perceptions observed should not be interpreted as encouragement for risky interactions or domesticating bats, as cross-species zoonotic transmission remains possible [36]. With regards to gender, both male and female learners demonstrated a strong willingness to learn more about bats. However, there was a significant knowledge gap, as over 50% of both genders disagreed that bats are important to the ecosystem. This highlights the need for education on the roles of species like bats in the environment. Gender also played a role in emotional perceptions, with females showing more negative attitudes towards bats. This aligns with previous studies suggesting that females tend to be more fearful or indifferent towards animals in general [66]. The authors attributed this to low knowledge about animals. Another study also revealed that boys show more positive attitudes toward bats and spiders than girls [21]. Despite this however, our study showed that less females agreed to negative behaviour towards bats suggesting that they did not view exterminating bats and eliminating them from the ecosystem as an ideal solution. This might suggest that females had more empathy towards bats [67,68]. The difference in preferences towards species has been documented in previous studies where gender plays a role in liking certain species of animals over others [31,69]. Females have been shown to be more likely to prefer more popular and neutral animals whilst males prefer less popular animals [69]. In this study, there was no significant impact of gender on believing in false myths about bats. Mystical beliefs of bats can lead to negativistic behaviour towards them [21]. Even though the study revealed that learners also believe in false myths about bats, this was about fifty percent of the learners in total in both males and females suggesting that not all the learners believed in the false myths.

Age was also found to significantly influence perceptions of students in the categories of scientistic and cognitive views. Older students were more enthusiastic to learn about bats, however they were more cognitively negative towards them. Another study found that older people had more positive attitudes towards bats [32] which is in line with our findings showing more cognitive negative perceptions toward bats by younger respondents. The impact of age however was not visible in the rest of the categories. This might have been due to the relatively small age difference amongst the learners.

One limitation of this study is the focus on a single, localized community, which may not fully capture the diversity of cultural or social beliefs about bats across other regions. Additionally, we assessed perceptions immediately following the intervention, leaving the long-term impact unexamined. Another limitation of the study was the inability to sample learners who were not part of the intervention to serve as a control group for comparison with those who participated in the intervention. Engagement efforts, such as posters and bat boxes, were accessible to all students, potentially influencing their perceptions and introducing biases for any learners who might have been designated as a control group within each school. Despite these limitations, the findings offer insights into the potential of educational interventions to reshape attitudes, particularly in communities where misinformation and myths about bats are prevalent. Future studies could build on these results by exploring longitudinal effects and testing similar interventions in diverse cultural settings to evaluate their scalability and adaptability.

## Conclusion

Our study demonstrated the usefulness of educational intervention as a tool in changing negative perceptions of learners towards bats. The extent to which perceptions were changed differed amongst the perception categories. With a more practical approach, it is likely that our intervention would have yielded much greater success. For species such as bats, awareness programs targeted at both young and adult individuals are crucial to promote these misconceived and misunderstood species.

## Supporting information

S1 Appendix The different categories and the statements that the standardized questionnaire comprised of. (DOCX)

S2 Appendix The standardized questionnaire that was used in the study. (DOCX)

S3 Appendix Descriptive statistics including gender composition, age distribution, and response distributions for all survey questions before and after the intervention. (DOCX)

S1 File Details of the children’s book used in the study. (DOCX)

## References

[1] Conover M. Resolving human wildlife conflicts: the science of wildlife damage management. J Wildl Manage. 2002;44(3).

[2] Peterson MN, Birckhead JL, Leong K, Peterson MJ, Peterson TR. Rearticulating the myth of human-wildlife conflict. Conserv Lett. 2010;3.

[3] Nyhus PJ. Human-Wildlife Conflict and Coexistence. Ann Rev Environ Resour. 2016;41.

[4] Redpath SM, Young J, Evely A, Adams WM, Sutherland WJ, Whitehouse A. Understanding and managing conservation conflicts. Trends Ecol Evol. 2013;28. 10.1016/j.tree.2012.08.021 23040462

[5] Siex KS, Struhsaker TT. Colobus monkeys and coconuts: a study of perceived human–wildlife conflicts. J Appl Ecol. 1999;36(6):1009–20. doi: 10.1046/j.1365-2664.1999.00455.x

[6] Dickman AJ. Complexities of conflict: The importance of considering social factors for effectively resolving human-wildlife conflict. Anim Conserv. 2010;13.

[7] Sjöberg L, Moen BE, Rundmo T. Explaining risk perception. An evaluation of the psychometric paradigm in risk perception research. Risk Analysis. 2004;10(2):665–72.

[8] Davis EO, O’Connor D, Crudge B, Carignan A, Glikman JA, Browne-Nuñez C. Understanding public perceptions and motivations around bear part use: A study in northern Laos of attitudes of Chinese tourists and Lao PDR nationals. Biol Conserv. 2016;203.

[9] Voigt CC, Kingston T. Bats in the Anthropocene: conservation of bats in a changing world. Springer Nature; 2016.

[10] Burgin CJ, Colella JP, Kahn PL, Upham NS. How many species of mammals are there? J Mammal. 2018;99(1).

[11] Walker’s bats of the world. Choice Rev Online. 1995;33(01).

[12] Boyles JG, Cryan PM, McCracken GF, Kunz TH. Economic importance of bats in agriculture. Science. 2011;332. 10.1126/science.1201366 21454775

[13] Kunz TH, de Torrez EB, Bauer D, Lobova T, Fleming TH. Ecosystem services provided by bats. Ann New York Acad Sci. 2011;1223. 10.1111/j.1749-6632.2011.06004.x 21449963

[14] Weier SM, Moodley Y, Fraser MF, Linden VMG, Grass I, Tscharntke T. Insect pest consumption by bats in macadamia orchards established by molecular diet analyses. Glob Ecol Conserv. 2019;18.

[15] Linden VMG, Grass I, Joubert E, Tscharntke T, Weier SM, Taylor PJ. Ecosystem services and disservices by birds, bats and monkeys change with macadamia landscape heterogeneity. J Appl Ecol. 2019;56(8):2069–78. doi: 10.1111/1365-2664.13424

[16] Taylor PJ, Grass I, Alberts AJ, Joubert E, Tscharntke T. Economic value of bat predation services – a review and new estimates from macadamia orchards. Ecosyst Serv. 2018;30.

[17] Jones G, Jacobs DS, Kunz TH, Wilig MR, Racey PA. Carpe noctem: The importance of bats as bioindicators. Endang Spec Res. 2009;8.

[18] Charbonnier Y, Papura D, Touzot O, Rhouy N, Sentenac G, Rusch A. Pest control services provided by bats in vineyard landscapes. Agric Ecosyst Environ. 2021;306.

[19] Maslo B, Mau RL, Kerwin K, McDonough R, McHale E, Foster JT. Bats provide a critical ecosystem service by consuming a large diversity of agricultural pest insects. Agric Ecosyst Environ. 2022;324.

[20] Nsengimana O, Walker FM, Webala PW, Twizeyimana I, Dusabe M-C, Sanchez DE, et al. Our good neighbors: Understanding ecosystem services provided by insectivorous bats in Rwanda. PLoS One. 2023;18(6):e0287536. doi: 10.1371/journal.pone.0287536 37352304 PMC10289311

[21] Prokop P, Tunnicliffe SD. “Disgusting” Animals: Primary School Children’s Attitudes and Myths of Bats and Spiders. Eurasia J Math Sci Tech Educ. 2008;4(2). doi: 10.12973/ejmste/75309

[22] Whitaker JO Jr, Douglas Lr. Bat Rabies in Indiana. J Wildlife Manag. 2006;70(6):1569–73. doi: 10.2193/0022-541x(2006)70[1569:brii]2.0.co;2

[23] Olnhausen LR, Gannon MR. An Evaluation of Bat Rabies Prevention in the United States, Based on an Analysis from Pennsylvania. Acta Chiropt. 2004;6(1):163–8. doi: 10.3161/001.006.0113

[24] Weber N, Nagy M, Markotter W, Schaer J, Puechmaille SJ, Sutton J. Robust evidence for bats as reservoir hosts is lacking in most African virus studies: A review and call to optimize sampling and conserve bats. Biol Lett. 2023;19. 10.1098/rsbl.2023.0358 PMC10646460 37964576

[25] Olival KJ, Weekley CC, Daszak P. Are bats really “special” as viral reservoirs? What we know and need to know. In: Bats and viruses: a new frontier of emerging infectious diseases. 2015.

[26] López-Baucells A, Rocha R, Fernández-Llamazares Á. When bats go viral: negative framings in virological research imperil bat conservation. Mamm Rev. 2018;48(1).

[27] Coleman JL, Randhawa N, Huang JCC, Kingston T, Lee BPYH, O’Keefe JM. Dying for décor: quantifying the online, ornamental trade in a distinctive bat species, Kerivoula picta. Eur J Wildl Res. 2024;70(4).

[28] Kamins AO, Restif O, Ntiamoa-Baidu Y, Suu-Ire R, Hayman DTS, Cunningham AA, et al. Uncovering the fruit bat bushmeat commodity chain and the true extent of fruit bat hunting in Ghana, West Africa. Biol Conserv. 2011;144(12):3000–8. doi: 10.1016/j.biocon.2011.09.003 22514356 PMC3323830

[29] Tanalgo KC, Sritongchuay T, Agduma AR, Dela Cruz KC, Hughes AC. Are we hunting bats to extinction? Worldwide patterns of hunting risk in bats are driven by species ecology and regional economics. Biol Conserv. 2023;279.

[30] Arrindell WA. Phobic dimensions: IV. The structure of animal fears. Behav Res Ther. 2000;38(5):509–30. doi: 10.1016/s0005-7967(99)00097-2 10816909

[31] Davey GC. Self-reported fears to common indigenous animals in an adult UK population: the role of disgust sensitivity. Br J Psychol. 1994;85(Pt 4):541–54. doi: 10.1111/j.2044-8295.1994.tb02540.x 7812671

[32] Musila S, Prokop P, Gichuki N. Knowledge and perceptions of, and attitudes to, bats by people living around Arabuko-Sokoke Forest, Malindi-Kenya. Anthrozoos. 2018;31(2).

[33] Mayen F. Haematophagous bats in Brazil, their role in rabies transmission, impact on public health, livestock industry and alternatives to an indiscriminate reduction of bat population. J Vet Med B Infect Dis Vet Public Health. 2003;50(10):469–72. doi: 10.1046/j.1439-0450.2003.00713.x 14720182

[34] Euren J, Bangura J, Gbakima A, Sinah M, Yonda S, Lange CE. Human interactions with bat populations in Bombali, Sierra Leone. Ecohealth. 2020;17(3). 10.1007/s10393-020-01502-y 33175278

[35] Jacobsen NHG, Du Plessis E. The Egyptian fruitbat Rousettus aegyptiacus as a problem animal in the Transvaal. Transvaal. 2023;35.

[36] Markotter W, Coertse J, De Vries L, Geldenhuys M, Mortlock M. Bat-borne viruses in Africa: a critical review. J Zoo. 2020;311. 10.1111/jzo.12769 PMC7228346 32427175

[37] Zhao H. COVID-19 drives new threat to bats in China. Science. 2020;367. 10.1126/science.abb3088 32217719

[38] Meyer M, Melville DW, Baldwin HJ, Wilhelm K, Nkrumah EE, Badu EK, et al. Bat species assemblage predicts coronavirus prevalence. Nat Commun. 2024;15(1). 10.1038/s41467-024-46979-1 PMC10994947 38575573

[39] Hassanin A, Tu VT, Pham PV, Ngon LQ, Chabane T, Moulin L, et al. Bat Rhinacoviruses Related to Swine Acute Diarrhoea Syndrome Coronavirus Evolve under Strong Host and Geographic Constraints in China and Vietnam. Viruses. 2024;16(7):1114. doi: 10.3390/v16071114 39066276 PMC11281452

[40] Lu M, Wang X, Ye H, Wang H, Qiu S, Zhang H. Does public fear that bats spread COVID-19 jeopardize bat conservation? Biol Conserv. 2021;254. 10.1016/j.biocon.2021.108952 PMC7837179 33518772

[41] Laverty TM, Teel TL, Gawusab AA, Berger J. Listening to bats: Namibian pastoralists’ perspectives, stories, and experiences. J Ethnobiol. 2021;41(1).

[42] Voigt CC, Kingston T. Bats in the Anthropocene. Springer International Publishing; 2016.

[43] Rakotomamonjy SN, Jones JPG, Razafimanahaka JH, Ramamonjisoa B, Williams SJ. The effects of environmental education on children’s and parents’ knowledge and attitudes towards lemurs in rural Madagascar. Anim Conserv. 2015;18(2).

[44] Williams ST, Williams KS, Constant N, Swanepoel L, Taylor PJ, Belmain SR, et al. Low‐intensity environmental education can enhance perceptions of culturally taboo wildlife. Ecosphere. 2021;12(7). doi: 10.1002/ecs2.3482

[45] Scrizzi A, Le Bel S, La Grange M, Cornélis D, Mabika C, Czudek R. Urban human-elephant conflict in Zimbabwe: a case study of the mitigation endeavour. Pachyderm. 2018;59:76–85. doi: 10.69649/pachyderm.v59i.83

[46] Pérez B, Álvarez B, Boso A, Lisón F. Design and Psychometric Properties of the BAtSS: A New Tool to Assess Attitudes towards Bats. Animals (Basel). 2021;11(2):244. doi: 10.3390/ani11020244 33498185 PMC7908982

[47] Nishimwe-Niyimbanira R. Income poverty versus multidimensional poverty: Empirical insight from Qwaqwa. Afr J Sci Technol Innovat Dev. 2019;12(5):631–41. doi: 10.1080/20421338.2019.1638585

[48] Mocwagae K, Mphambukeli T, Magaiza G, Hansen M, Otomo P, Delves JL. Planning for effective and sustainable water access and provision in QwaQwa through the UN sustainable development goals. Sustainable futures in Southern Africa’s mountains: Multiple perspectives on an emerging city. Cham: Springer International Publishing; 2023. p. 105–26.

[49] Statistics South Africa. Census. Local Municipality | Statistics South Africa. 2022. Available from: www.statssa.gov.za/?page_id=993&id=maluti-a-phofung-municipality

[50] Slater R. Differentiation and diversification: Changing livelihoods in Qwaqwa, South Africa, 1970-2000. J South Afr Stud. 2002;28(3).

[51] Tsotetsi CT, Omodan BI. Transforming socio-economic development in QwaQwa community of South Africa. Int J Res Bus Soc Sci. 2022;11(3):184–94. doi: 10.20525/ijrbs.v11i3.1762

[52] Monadjem A, Taylor PJ, Schoeman MC. Bats of southern and central Africa: a biogeographic and taxonomic synthesis. Wits University Press; 2020.

[53] Christensen RHB. Cumulative link models for ordinal regression with the R package ordinal. J Stat Software. 2018;35.

[54] R Core Team. R: A language and environment for statistical computing. R Foundation for Statistical Computing; 2019.

[55] Burnham KP, Anderson DR. Multimodel inference: Understanding AIC and BIC in model selection. Soc Methods Res. 2004;33.

[56] Johnson JB, Omland KS. Model selection in ecology and evolution. Trends Ecol Evol. 2004;19. 10.1016/j.tree.2003.10.013 16701236

[57] Wickham H. Programming with ggplot2. 2016.

[58] Cohen MR. Environmental Information Versus Environmental Attitudes. J Environ Educ. 1973;5(2):5–8. doi: 10.1080/00958964.1973.10801804

[59] Bradley JC, Waliczek TM, Zajicek JM. Relationship Between Environmental Knowledge and Environmental Attitude of High School Students. J Environ Educ. 1999;30(3):17–21. doi: 10.1080/00958969909601873

[60] Mangas VJ, Martinez P, Pedauyé R. Analysis of Environmental Concepts and Attitudes Among Biology Degree Students. J Environ Educ. 1997;29(1):28–33. doi: 10.1080/00958969709599104

[61] Kaninsky M, Gallacher S, Rogers Y. Confronting people’s fears about bats: Combining multimodal and environmentally sensed data to promote curiosity and discovery. In: DIS 2018 - Proceedings of the 2018 Designing Interactive Systems Conference. 2018.

[62] Johnson L, Price EC. Battitude: A virtual zoo “bat experience” produces positive change in attitudes to an unpopular species. J Zoo Aquar Res. 2023;11(1).

[63] Gili F, Bertolino S, Rolando A. Using mobile device built-in microphones to monitor bats: a new opportunity for large-scale participatory science initiatives. Biodivers Conserv. 2024;33(5):1623–43.

[64] Tanalgo KC, Catherine Hughes A. The potential of bat-watching tourism in raising public awareness towards bat conservation in the Philippines. Environ Challeng. 2021;4:100140. doi: 10.1016/j.envc.2021.100140

[65] Pennisi LA, Holland SM, Stein TV. Achieving Bat Conservation Through Tourism. J Ecotour. 2004;3(3):195–207. doi: 10.1080/14664200508668432

[66] Kellert SR, Berry JK. Attitudes, knowledge, and behaviours toward wildlife as affected by gender. Wildlife Society Bulletin. 1987;15(3).

[67] Herzog HA, Betchart NS, Pittman RB. Gender, sex role orientation, and attitudes toward animals. Anthrozoos. 1991;4(3).

[68] Taylor N, Signal TD. Empathy and attitudes to animals. Anthrozoos. 2005;18.

[69] Bjerke T, Østdahl T. Animal-related attitudes and activities in an urban population. Anthrozoos. 2004;17.

