# Supplementary material for: Environmental education positively impacts the perceptions of learners towards bats in schools in a low socio-economic area in South Africa
Source: PLoS One. 2025 Dec 19;20(12):e0335652. doi: 10.1371/journal.pone.0335652 (PMC12716788; doi:10.1371/journal.pone.0335652)
Supplement: S2 Appendix — (DOCX) [file pone.0335652.s002.docx]

**S2 Appendix:** The standardized questionnaire that was used in the study.

**Design and Psychometric Properties of the BAtSS: A New Tool to Assess Attitudes towards Bats - Perez et al. 2021**

Bat Education and Citizen Science Project

Coordinator: Veli Monday Mdluli

Name of School:…....................................................

Age of participant: ………………………………… Gender of participant:……………

Questionnaire questions to be translated if necessary and answered on a scale of 1 to 5

| **Scientistic** | **1 – strongly disagree** | **2 – disagree** | **3 - neutral** | **4 - agree** | **5 – strongly agree** |
| --- | --- | --- | --- | --- | --- |
| 1 I would like to learn more about bats |  |  |  |  |  |
| 2 Knowing about the activity of bats is important for me |  |  |  |  |  |
| 3 I would like to take part in a trip or a congress, or other activity, to  learn about bats. |  |  |  |  |  |
| 4 It would be interesting to take part in a scientific activity about bats |  |  |  |  |  |
| 5 I would like to exchange knowledge about bats with other people |  |  |  |  |  |
| 6 It would be interesting to be able to teach others about bats |  |  |  |  |  |
| 7 I would like to read a scientific article or see a documentary  about bats |  |  |  |  |  |

| **Positivistic** | **1 – strongly disagree** | **2 – disagree** | **3 - neutral** | **4 - agree** | **5 – strongly agree** |
| --- | --- | --- | --- | --- | --- |
| 8 Bats are important for the functioning of our ecosystem |  |  |  |  |  |
| 9 Humans should protect bats |  |  |  |  |  |
| 10 Spaces should be set aside for bat conservation in farmland |  |  |  |  |  |
| 11 Humans must learn to coexist with bats |  |  |  |  |  |
| 12 Bat excrement is a source of good fertiliser for farming |  |  |  |  |  |
| 13 Bats help in the biological control of pests |  |  |  |  |  |
| 14 Bats help food security |  |  |  |  |  |
| 15 Some species of bat help to disperse tree seeds |  |  |  |  |  |
| 16 The activity of bats gives added value to crops in the market |  |  |  |  |  |

| **Emotional Negativistic** | **1 – strongly disagree** | **2 - disagree** | **3 - neutral** | **4 - agree** | **5 – strongly agree** |
| --- | --- | --- | --- | --- | --- |
| 17 Bats are ugly |  |  |  |  |  |
| 18 Bats are dangerous for humans |  |  |  |  |  |
| 19 I am afraid of bats |  |  |  |  |  |

| **Behavioral Negativistic** | **1 – strongly disagree** | **2 - disagree** | **3 - neutral** | **4 - agree** | **5 – strongly agree** |
| --- | --- | --- | --- | --- | --- |
| 20 Bats should be exterminated |  |  |  |  |  |
| 21 We should attack bats |  |  |  |  |  |
| 22 Bat refuges should be eliminated to prevent them from breeding (block up caves, cut down trees, etc.) |  |  |  |  |  |
| 23 We should stop bats from reproducing |  |  |  |  |  |

| **Cognitive Negativistic** | **1 – strongly disagree** | **2 - disagree** | **3 - neutral** | **4 - agree** | **5 – strongly agree** |
| --- | --- | --- | --- | --- | --- |
| 24 Bats’ activity contaminates crops |  |  |  |  |  |
| 25 Bats attract other species of rodents |  |  |  |  |  |
| 26 Bats can be dangerous for domestic animals |  |  |  |  |  |
| 27 Bats contaminate water resources |  |  |  |  |  |
| 28 Bats damage machinery/buildings |  |  |  |  |  |
| 29 Bats harm agriculture |  |  |  |  |  |
| 30 Bats are aggressive |  |  |  |  |  |

| **Myths** | **1 – strongly disagree** | **2 - disagree** | **3 - neutral** | **4 - agree** | **5 – strongly agree** |
| --- | --- | --- | --- | --- | --- |
| 31 The bat is a symbol of ill omen |  |  |  |  |  |
| 32 When you see a bat, it is a sign that someone wants to harm you |  |  |  |  |  |
| 33 Bats become vampires |  |  |  |  |  |
| 34 Bats should be burnt to prevent witchcraft |  |  |  |  |  |
| 35 Do you think bats are responsible for the current COVID-19 pandemic? |  |  |  |  |  |
