# Supplementary material for: Environmental education positively impacts the perceptions of learners towards bats in schools in a low socio-economic area in South Africa
Source: PLoS One. 2025 Dec 19;20(12):e0335652. doi: 10.1371/journal.pone.0335652 (PMC12716788; doi:10.1371/journal.pone.0335652)

S3 Appendix: Descriptive statistics including gender composition, age distribution, and response distributions for all survey questions before and after the intervention.

Appendix 3a. Distribution of learners by gender across intervention sessions. Values are presented as percentages, with actual numbers in brackets, for the pre-intervention (Before), post-intervention (After), and combined (Total) samples.

| Intervention | Female | Male |
| --- | --- | --- |
| Before | 52.5% (149) | 47.5% (135) |
| After | 57.4% (109) | 42.6% (81) |
| Total | 54.4% (258) | 45.6% (216) |

Appendix 3b: Summary of learner age distributions by intervention session. The table shows the number of learners (N), mean age, standard deviation (SD), minimum, and maximum ages for the pre-intervention (Before) and post-intervention (After) surveys.

| Intervention | N | Mean Age | SD Age | Min Age | Max Age |
| --- | --- | --- | --- | --- | --- |
| Before | 290 | 12.7 | 1.43 | 11 | 18 |
| After | 201 | 12.5 | 1.14 | 11 | 16 |

Appendix 3c. Boxplot showing the distribution of learners’ ages before and after the educational intervention. The median, interquartile range, and overall spread of ages are illustrated for each intervention group.


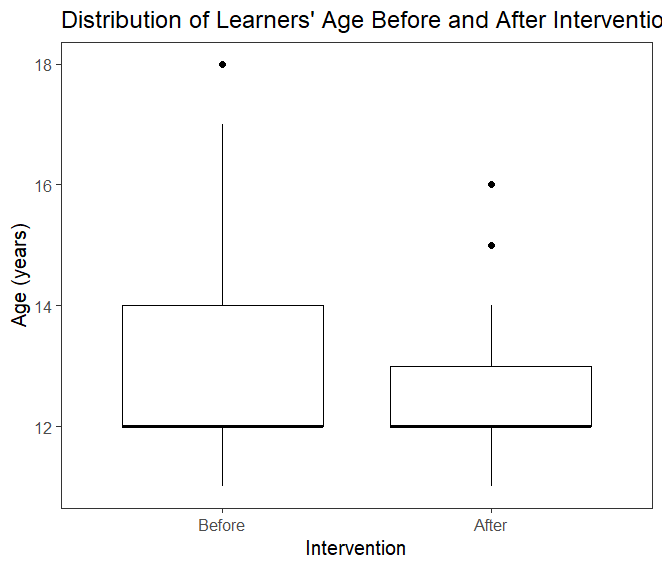


Appendix 3d: Proportion of learner responses (“Disagree,” “Neutral,” and “Agree”) to the different questions in each category before and after the educational intervention. Questions are labelled Q1 to Q35 and the bars show the distribution of responses across the two intervention sessions.


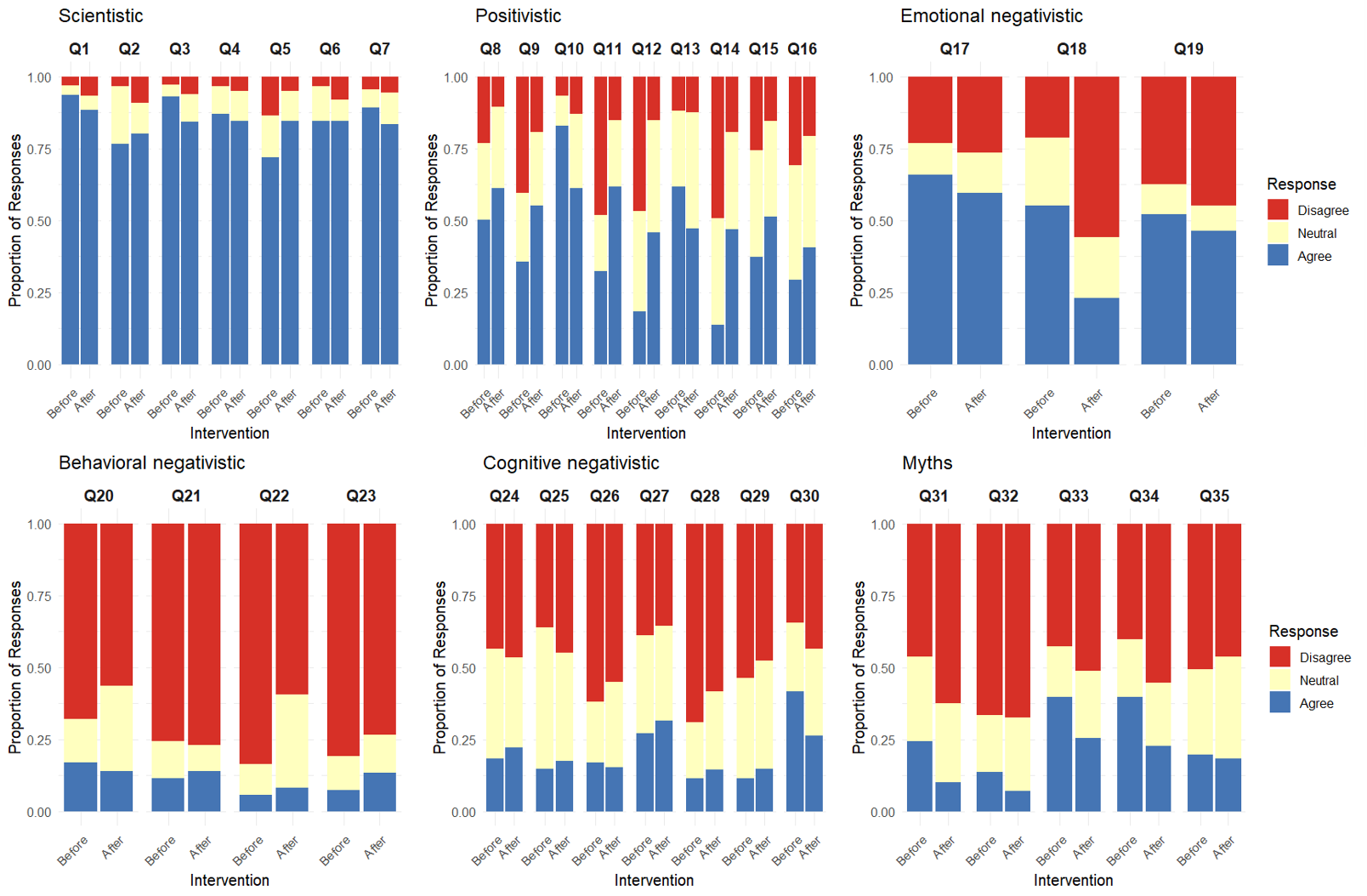

Supplement: S3 Appendix — (DOCX) [file pone.0335652.s003.docx]
