# Supplementary material for: Environmental education positively impacts the perceptions of learners towards bats in schools in a low socio-economic area in South Africa
Source: PLoS One. 2025 Dec 19;20(12):e0335652. doi: 10.1371/journal.pone.0335652 (PMC12716788; doi:10.1371/journal.pone.0335652)
Supplement: S1 File — (DOCX) [file pone.0335652.s004.docx]

**S1 File**

Details of the children’s book used in the study

Title: **QwaQwa’s Heroes After** **Sunset**

ISBN: 978-1-7764559-6-6

Summary

After getting lost on top of the QwaQwa Mountain, a ten-year-old boy becomes friends with a bat that helps him navigate his way down the mountain and back home. The boy grows passionate about bats and their conservation. With the help of his science teacher, he starts giving awareness talks at his school, but this is met by heavy backlash from some of the community elders who believe that bats are a symbol of ill omen which results in the boy and teacher almost getting suspended for showing a live bat to students. It takes a great courageous effort by the bats and the Chief of QwaQwa to finally convince everyone that the bats are important for the well-being of both the community and ecosystem.

**Book Reviews**

**Please see the reviews below from several people who provided helpful advice:**

“I’ve just finished reading Mondays book and it is a real tearjerker.  Such a beautiful story so imaginatively written and will be a fascinating read for any child, never mind a wonderful learning experience. The whole story is so vivid, with details and descriptions to capture the imagination of any child.  Congratulations to Monday!  You do need the right beautiful illustrations to complete this book which will be one for young people to treasure forever!”

- Ms Felicity Keats of Umsinzi Publishers

“This is a story of fiction based on real scientific facts to try change the minds of children regarding the negative perceptions that many South African have of bats.

The book is a great mix of fantasy and educational for both young and old with easy-to-relate-to characters as I appreciate the use of the local South African names, setting and location. I hope the relevant location of being set in Qwaqwa will be relatable to the students and even more so when translated into multiple languages.

I think the pace is fast enough to illustrate the themes of growing up, helping your community and that anyone can make a difference to protect our heritage. Although I sometimes find the writing style repetitive or long, it is clear enough and appropriate for the targeted age group. I would recommend a glossary list of words that might be unfamiliar to scholars who aren’t English-speaking as a first language.

Just as a small opinion, I do wonder if some of the tough socio-economic references are perhaps not appropriate for the age group however, the overall impact of the book is very inspiring and motivating! With the addition of illustrations from the scholars in Qwaqwa, I have no doubt this will be a great book for environmental education in South Africa.

Well done Mr Mdluli!”

- Alexandra Howard of the Mountain Bat Lab at UFS (PhD student)

“The story addresses most of the beliefs that our local people have about bats. It is a great book to teach young children and even adults about how much valuable bats are in the environment. I like that the story has been localized to Qwaqwa as it makes it easier for the reader to relate to the characters. The way the story has been written is also easy to follow and great job on having a seSotho version of this story. As a teacher, I too have learned things about bats which I did not know and hence I believe that this book is very valuable in changing the ways that people see bats and how they think of them. I wish that more work like this can be done in our region to empower our young people to take better care of all animals and the environment. “

- Tsietsi Ntlangoe, Teacher at Mamello Primary School
